# Spatial Evaluation of Oral Lichen Planus Patients Referred to Shiraz Dental School, Iran: a Medical Geography Approach

**DOI:** 10.30476/DENTJODS.2021.90760.1519

**Published:** 2022-09

**Authors:** Maryam Zahed, Ali Goli, Elaheh Zamirian, Saeid Zahed, Azita Azad, Fatemeh Aghaei

**Affiliations:** 1 Oral and Dental Disease Research Center, Dept. of Oral and Maxillofacial Medicine, School of Dentistry, Shiraz University of Medical Sciences, Shiraz, Iran; 2 Dept. of Sociology and Social Planning, College of Economics, Management and Social Sciences, Shiraz University, Shiraz, Iran; 3 Undergraduate Student, Student Research Committee, School of Dentistry, Shiraz University of Medical Sciences, Shiraz, Iran; 4 Postgraduate Dept. of Restorative, School of Dentistry, Shiraz University of Medical Sciences, Shiraz, Iran

**Keywords:** Medical Geography, Dentists, Spatial Distribution, Oral Lichen Planus

## Abstract

**Statement of the Problem:**

Oral lichen planus (OLP) is an important inflammatory disease concerning its tendency to malignancy. The etiopathogenesis of this disease is still unknown. Medical geography uses geographic techniques to study factors related to location that cause uneven distribution of disease.

**Purpose:**

This study was conducted to map OLP in patients referred to Shiraz Dental School with medical geography techniques and investigate any possible relationship between the number of dentists and health-centers in different regions of Fars province with the number of referrals.

**Materials and Method:**

In this cross-sectional study, we evaluated the records of OLP patients referred to Shiraz Dental School from 2007 to 2018. Age, sex, place of residence, occupation, level of education, location of involvement, duration, and cutaneous involvement were recorded. The number of dentists and healthcare centers was obtained from the statistical records of Fars province. A Geographic Information System was used for the assessment of the spatial distribution of OLP. Ordinary least squares (OLS) and geographically weighted regression (GWR) indices were used for evaluating the relationship between the number of dentists and health centers with the number of referrals.

**Results:**

From 1006 records, 457 were studied, from which 71% were female (age range of 41-60). The regression coefficient was 0.937 for the number of dentists in each county compared to OLP referrals indicating a strong relationship. The regression coefficient was 0.983 for the number of health-centers. According to GWR analyses in Shiraz neighboring counties like Marvdasht, Sepidan, and Sarvestan, there is a positive relationship between the number of dentists and OLP patients.

**Conclusion:**

The results revealed that dentists and health care centers had a good cognition of referring patients with OLP (as a premalignant lesion) for definite diagnosis in Fars counties. But both groups need more education in this matter. Moreover, referrals from settlements near Shiraz were more common because of their easier access.

## Introduction

Oral lichen planus (OLP) is an important chronic mucocutaneous disease with unknown etiology that affects 0.5 to 2.2% of the population. The mean age of patients reported in the literature is 55 years and it is more prevalent in women [ 1 - 5 ]. OLP is mostly a white keratotic lesion with various clinical forms, including reticular, popular, or plaque-like. These forms can be accompanied by ulcers, pigmentation, bullae, or erosions [ 1 ]. Oral lesions may be seen in any site of ​​the oral mucosa, but the buccal mucosa, dorsal surface of the tongue, and gingiva are the most common places. Moreover, OLP is commonly observed as a bilateral lesion that involves multiple sites of the oral mucosa [ 4 ]. 

Although the exact etiology of this disease remains unclear, alterations in the cell-mediated immune response play a central role in its pathogenesis. Therefore, each factor that attenuates the immune system can contribute to the disease [ 3 , 6 - 10 ]. The role of mental disorders, especially depression and anxiety are debated among the probably contributing factors [ 7 , 11 - 12 ]. 

OLP is an imperative disorder concerning its malignant potential [ 1 - 2 , 13 ]. Additionally, the definite treatment of this disorder remains a challenge for clinicians [ 14 - 15 ]. Lichenoid reactions are similar clinical and histopathological entities where an etiologic factor such as a dental filling or a certain medication can be attributed to the presence of the disease [ 16 ]. 

Medical geography also called health geography is a branch of medical research that utilizes geographical techniques to study the health and distribution of diseases around the world. In addition, medical geography investigates the effects of climate and habitat on the health of a certain population [ 17 ].

There are previous studies that investigated the spatial prevalence of other diseases like mental disability, tuberculosis, breast, and prostate cancer [ 18 - 21 ]. However, no similar study has been published on lichen planus disease and its geographical distribution in any part of the world.

This study aimed to investigate OLP disease in patients referred to Shiraz Dental School from different regions of Fars province, Iran, with a medical geographical perspective. Also, we compared the rate of OLP referrals to this center from different locations regarding the number of dentists and health centers in each region.

## Materials and Method

In this cross-sectional study, all records of the Department of Oral and Maxillofacial Medicine of Shiraz Dental School from 2007 to 2018 were evaluated. After reviewing the records, the files with definitive clinical or histopathological diagnosis of OLP were enrolled in the study, and incomplete records, without address and definitive diagnosis were excluded.

This study was approved by the Ethical Committee of Shiraz University of Medical Sciences, IR.SUMS.DENTAL.REC.1398.018.

A total of 1006 records of OLP subjects were collected. Subjects with a definite diagnosis of OLP who had a registered telephone number were chosen and after contacting the patients an oral informed consent was taken. Data including age, sex, city of residence, education, occupation, the form of OLP, duration of involvement, and recovery of lesions were collected from the medical records. Missing data was obtained whilst contacting the patients. Information regarding the number of dentists and health centers was collected from the Fars Province Statistical Yearbook of 2017 Chapter-seventeen through the National Statistics Portal.

To seek the geographical distribution of OLP in Fars province during 2007-2018 Geographic Information System software was used. The Geographic Information System as a spatial analytical tool can provide a more realistic point of view to represent and analyze phenomena where spatial distribution is important [ 18 ].

To investigate data homogeneity and heterogeneity ordinary least squares (OLS) was used as regression indices, and its general equation is as follows (1-1):

$\hat{y}=a+\mathrm{bx}$ (1.1)

Where $\hat{y}$ is the predictive value of the dependent variable, α is the regression coefficient or intercept elevation, ^b^ is the regression coefficient, and *X* is the independent coefficient value. It should be noted that the predictive value of $y(\hat{y})$ is not necessarily measured accurately since there is usually an error between the true and the predictive values. The true value of expresses as follows: 

y=a+bx+ϵ (2-1)

Where *ɛ* represents the error between *y*, and $\hat{y}$ . Multivariate regression including independent variables and coefficients are added to the equation as follows:

$y=a+\mathrm{bx}+b_{1}x_{1}+b_{2}x_{2}+b_{3}x_{3}+\epsilon$ (3-1)

The constraints of OLS regression are their inability to consider the spatial differences in variables. Therefore, to understand patterns of association between two or more variables in a region, it is important to consider the impact of the contingency of related values for adjacent regions of space. One of the methods of controlling the impact of spatial correlation is geographically weighted regression (GWR) [ 18 ].

The basis of the GWR is that each regression point (*i*) has a degree of impact around *i* which is described by the spatial weighting function; such that the observations close to *i* have more effect than the farther points in estimating parameters [ 22 ]. To describe this model, general regression must be used as follows: 

$y_{i}=\beta_{0}+\sum_{k}\beta_{k}x_{\mathrm{ik}}+\epsilon$

As shown *y* is the estimated value for the dependent variable of the *i* observation, *β_0_* intercepts elevation, *β_k_* is the parameter estimation for the *k* variable, *x_ik_* is the value of *k_th_* for the *i* variable, *ɛ_i_* and is the disturbance component [ 23 ]. The general model measures a single regression equation for all observations, whereas the GWR constructs a separate regression equation for each observation and each equation is determined by the different weight of observations over the dataset [ 24 ].

As noted above, the GWR revises the classical regression model as follows: 

$y=\beta_{0}(u_{i},v_{i})+\sum_{k}\beta_{k}(u_{i},v_{i})x_{\mathrm{ik}}+\epsilon_{i}$

Where:

*y*= dependent variable observations in site *i*

*x_ik_* = Observations of the *k^kt^* independent variable in site,*i*

*β_ik_*=*k^th^* Variable coefficient in site *i*

$\left(u_{i},v_{i}\right)$ = site coordinates of *i*

*ɛ_i_* = Disruption component in site *i*

As mentioned above, in order to measure the $y=\beta_{0}(u_{i},v_{i})+\sum_{k}\beta_{k}(u_{i},v_{i})x_{\mathrm{ik}}+\epsilon_{i}$ equation, data closer to the site *i* in $\beta_{k}(u_{i},v_{i})$ estimation are more weighted than farther away of *i*. In fact, the equation measures the relationships around each *i* site. Hence, weighted least square provide the principles to understanding how a GWR works [ 22 ]. In a GWR, an observation is weighted based on its proximity to the site *i* so the weight of an observation is not constant in space and varies with concern to location. Data close to observations weigh more than observations farther away. Therefore: 

$\hat{\beta }(u_{i},v_{i})={\left(X^{T}W(u_{i},v_{i})X\right)}^{-1}X^{T}W(u_{i},v_{i})y$

Therefore, $\hat{\beta }$ expresses estimation, $W(u_{i},v_{i})$is a n×n matrix whose elements are zero outside a diagonally bordered band and band matrix represents the geographical weight of the n observed data to *i* point regression.

To explain more clearly, consider the classic regression equation in the form of a matrix: 

Y=Xβ+ϵ

as the parameter vector is estimated,β is constant in space and can be estimated by: $\hat{\beta }={\left(X^{T}X\right)}^{-1}X^{T}Y$

GWR equals: Y=(β⊗X)1+ϵ

Therefore, ⊗ is the logical multiplication operator in each *β* entry that is multiplied by the corresponding element *x*. According to the above, spatial relationship modeling through Arc Geographic Information System was used for GWR analysis and OLS. Also, statistical analysis was performed by SPSS software (version 2018). All quantitative data were presented as mean±SD and percentage. 

## Results

From 1006 records of OLP subjects, which were collected from the Oral, and Maxillofacial Medicine department of Shiraz University of Medical Sciences, 86 were incomplete and were excluded from the study. Of the remaining 920 patients, only 546 responded to our contact and participated in the study, ultimately 457 belonged to Fars Province, and the rest were related to other cities in the country that were excluded from our study. Of these 457 patients with OLP, 3 patients had passed away and 2 were suffering from oral squamous cell carcinoma. The characterizations of patients according to the place of residence are given in Table 1. Overall, OLP was more frequent in women, where 70.9% of patients were female and 29.1% were male. The mean age of the patients was 48.96± 12.8. The majority of patients were referred to Shiraz Dental Clinic in less than 6 months from the onset of lesions (49.4%). The least number of patients belonged to over 5 years of disease onset group upon referral (6.6%). Statistical analysis showed that the majority of patients had a high school diploma (64.46%). Meanwhile more than half of the patients (71.11%) had no skin involvement and most patients (48.47%) had mild to moderate severity spectrum. Furthermore, less than a quarter of patients (about 15%) had lichenoid type disease, not OLP. The buccal mucosa and after that the gingiva (buccal: 1092/379 and gingival: 1092/233) were the most sites of OLP involvement.

**Figure 1:**
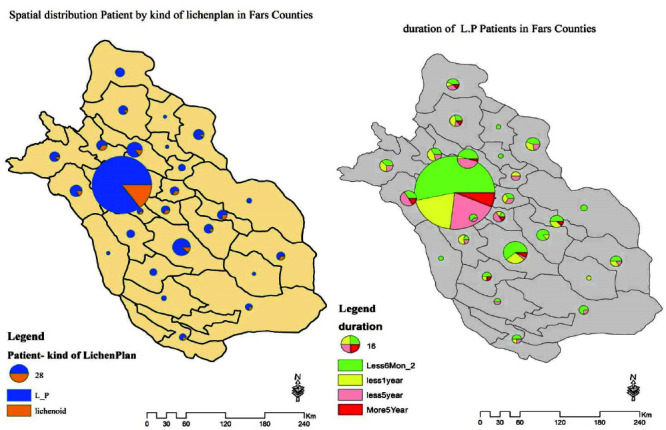
Distribution of disease based on oral lichenoid reaction or oral lichen planus type (left) and duration of disease involvement before referring to Shiraz Dental Clinic (Right)

Fars province contains 29 counties in 2020 with about 4.853.000 population. Geographical distribution showed that more than half of the patients with OLP and lichenoid reaction in Fars province never completely recovered from the disease. The number of patients with squamous cell carcinoma was two and the death rate was very low. OLP was more prevalent than lichenoid reactions in most counties of Fars province. The geographic map shows that the duration of OLP involvement before referral to Shiraz Dental School was 6 months or less in the majority of patients of most counties (Figure 1).

**Table 1:** Frequency of OLP patients by gender, education and occupation and number of dentists and health centers in different counties of Fars Province

County	Sex		Occupation			Level of education			Dentists	Health Centers
	Male	Female	Housewife	Employed	Not employed	Academic	Diploma	Illiterate		
Abadeh	6	1	6		1	3	4		3	11
Estahban	6	2	5	3		1	6	1	4	9
Eghlid	5	2	4	2	1	4	2	1	4	16
Jahrom	16	10	12	12	2	9	15	2	17	35
Darab	4	2	4	1	1	2	4		11	22
Sepidan	5	4	5	3	1	3	6		7	13
Shiraz	206	88	152	94	48	80	192	22	73	213
Fasa	6	1	5	2		1	6		16	22
Firoozabad	4	1	3	2		2	2	1	5	11
Kazeroon	9	3	9	3		1	10	1	12	27
Larestan	3	1	3	1			4		21	31
Marvdasht	18	2	15	4	1	3	13	4	17	39
Mamasani	6	2	4	2	2	1	5	2	6	19
Neyriz	1	1		1	1	2			6	16
Lamerd	3	1	3				3	1	4	14
Bavanat	6	3	6				9		2	9
Arsanjan	1	3	1			1	3		2	5
Khorambid	1		1				1		4	6
Zarin dasht	1		1				1		3	7
Ghirokarzin	3	1	3				4			9
Mohr									1	11
Farashband		1					1		3	6
Pasargad		1					1		3	3
Khonj	2		2					2	7	8
Sarvestan	6		6				3	3	3	4
Rostam									3	7
Gerash									4	4
Kavar	3		3				3		2	11
Kharameh	3	3	3	1	2	2	4		5	9
Total	133	324	256	60	141	116	301	40	248	597

The regression coefficient of the relationship between the number of dentists in each county of Fars province and the number of patients referred from that city with oral lichen planus was equal to 0.937, which indicates a strong relationship between the two variables. The square of the regression coefficient was 0.87 which indicates a change of 0.87 units per client per each unit change in the number of dentists. This regression was 0.983 for the number of health centers and the number of referrals. The square of the regression coefficient was 0.96 which indicates a change of 0.96 units per client per each unit change in the number of health centers.

**Figure 2:**
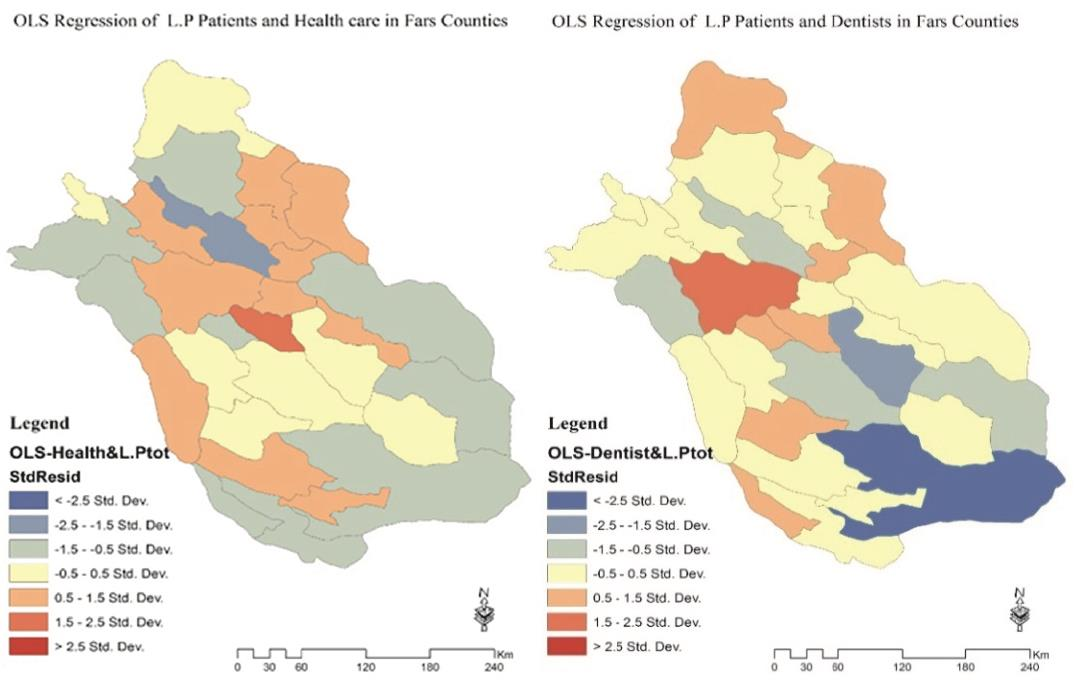
The relationship between the number of dentists and health centers in each county with the number of lichen planus (L.P) patients referred to Shiraz Dental School according to ordinary least square (OLS), standard residual (StdResid)

**Figure 3:**
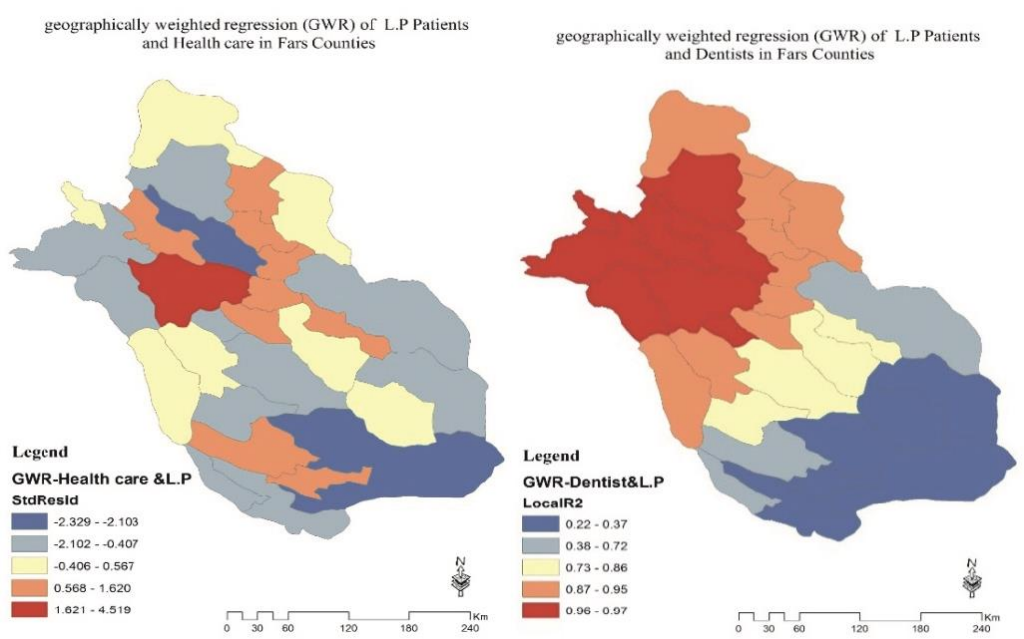
The relationship between the number of dentists and health centers in each county and the number of patients with lichen planus (L.P) referred to Shiraz Dental School according to geographically weighted regression (GWR), standard residual (StdResid)

In the 29 counties surveyed, the regions marked by the red-orange color spectrum have a direct relationship between the two variables (red is associated with a stronger relationship), and the regions with the blue gray color spectrum have an inverse relationship between the two variables. Regions marked in yellow represented significant relationship between the two variables. According to GWR, cities like Marvdasht which are near Shiraz have a strong relationship between the number of dentists and the number of OLP referrals, but this was not true for the number of health centers in this county. Also, cities like Larestan which are located in the south of the province have an inverse relationship in the number of dentists and health centers with the number of referrals (Figures 2 and Figure 3).

## Discussion

The overall results of this study revealed that OLP was more prevalent in females 41-60 years of age. Skin involvement was not common in OLP patients referred to Shiraz Dental School and most patients had a high school diploma. The majority of referrals were under 6 months of lesion development. Moreover, a strong relationship was seen between the number of dentists and the number of health centers in each county of Fars province with the number of OLP patients referred to the specialized treatment center in Shiraz Dental School. This means that with the increase in the number of dentists and health centers, the number of referrals had increased. 

The relationship between the number of dentists and health centers with the number of OLP patients in each county was primarily assessed by the OLS method. The direct relationship between the number of dentists and referrals like what is seen in Shiraz and Abadeh and the number of health centers and referrals like Sarvestan (Figure 2) may indicate that dentists or caregivers in these regions were aware of OLP diagnosis and referred these patients to the principle treatment center of Shiraz Dental School. The inverse relationship between the number of dentists and health centers and the number of patients referred, like what is seen in Larestan, shows that either the number of dentists was high and the number of referrals to Shiraz was low or the number of dentists was low and the number of referrals to Shiraz was high. The reason for this can be related to the distance between these counties and Shiraz. Additionally, the reason for fewer referrals to Shiraz dental school in an inverse relationship can be the presence of qualified dentists and caregivers in treating OLP in these cities. Furthermore, the reason for more referrals can be the lack of sufficient dentists and the lack of facilities in these cities. 

It should be noted that lichen planus is a mucocutaneous disease, which can be diagnosed by specialists such as dermatologists, ENT specialists, rheumatologists, and dentists concurrently. Therefore, in cities where there was no significant relationship between the two variables like Firoozabad, patients may have been referred to other specialists (Figure 2).

Subsequently, the GWR method was performed to investigate the relationship between the number of dentists and health centers in each county and the number of referrals. As previously mentioned, in this method, the effect of proximity and neighboring on the rate of patients' referral is considered. As it is shown in Figure 3, the data is presented as a cluster. Therefore, the province is divided into three general categories regarding the number of dentists and patients:

1. Cities with a high number of dentists and a high number of referrals marked as red "Hot spots" (Abadeh, Marvdasht, etc.). Shiraz proximity and accessibility is the main cause for this strong relationship. 

2. Cities with a high number of dentists and low referrals marked as blue "Cold spots" (Larestan, Lamerd, Darab). This is due to the distance, inaccessibility, and limited facilities of these cities. 

3. Cities with low dentists' rates as well as low referrals, in which OLP patients were probably treated by other specialists and they did not seek dental treatment.

Likewise, the number of health centers and the number of referrals using the GWR method are seen in figure 3. The only difference with the previous situation is that no cluster-state is seen. It is speculated that most patients were referred by dentists rather than physicians or health caregivers and also patients who live in cities near Shiraz tend to seek treatment in Shiraz centers.

González‐Moles *et al.* [ 6 ] recently published a study on the worldwide prevalence of OLP. In this systematic review, they reported that the global pooled prevalence of OLP was 1.01%, and they observed a marked geographical difference. The highest prevalence was in Europe and the lowest in Asia. They found that oral medicine/oral pathology specialists report significantly higher prevalence (1.80%) than dentists (0.61%) and dermatologists (0.33%; *p*< .001). Therefore, they concluded that dentists and dermatologists need continuing education regarding OLP and reliable diagnostic criteria must be defined for better diagnosis. These findings confirm our results for the need to raise awareness about this disease in health care workers. 

In the study conducted by, Goli *et al.* [ 18 ] the same OLS and GWR regression indices were used to investigate the spatial prevalence of mental disability and the related social and demographic factors in Iran. They concluded that the use of spatial geography in conjunction with medical research helps understand the effect of location on health issues and also helps promote population health. But OLP has not been previously investigated in this manner. 

The data analysis also showed that the majority of patients referred to Shiraz Dental School were suffering from oral lesions less than 6 months. It is worthy to say that the number of men who were referred was about 2.2 times more than the number of women during this period. A correct and early diagnosis of oral lesions is the main key to successful treatment [ 16 ]. Moreover, the majority of patients (51.85%) had mild disease. Therefore, this shows the benefits of early diagnosis and referral and also the awareness of health care providers and dentists, in referring these patients. OLP is generally regarded to represent a potentially malignant disorder in the oral mucosa. Therefore, early referral of patients and proper diagnosis by trained specialists prevent further complications [ 1 ]. In a previous study, 1.03% of OLP patients showed malignancy potentials in Shiraz, Iran [ 25 ]. In another study, two out of 64 (3.12%) patients had squamous cell carcinoma during a two-year follow-up [ 26 ]. In a recent systematic review and meta-analysis, 0.44% of OLP patients were considered to have undergone malignant transformation [ 2 ].

In addition, data analysis in terms of education and occupation showed that most patients had a high school diploma or lower education, and were mostly housewives. Previous studies have shown that stress, anxiety, and depression are among the initiating factors of various autoimmune reactions that have been implicated in the pathogenesis of lichen planus disease [ 11 - 12 ]. Varghese *et al.* [ 27 ] reported that 57% of patients with erosive lichen planus had a history of stress. Thus, the increasing prevalence of OLP in women and its association with lower education levels may be related to financial dependence, the emergence of psychological pressures and low levels of awareness for coping with stress.

It is worthy to note that Shiraz Dental School was the exclusive center in the south of the country to study and treat this disease and other mucocutaneous lesions during these years [ 28 ]. Therefore, we can claim that the data collected in this study is a high percentage of OLP patients seeking treatment in this region. However, due to the slow development of the disease, many people are not diagnosed promptly; therefore, the statistical population in this study does not represent all patients with OLP in Fars province. And the incidence of this disease cannot be measured because we do not have the exact number of patients in the whole province. 

It is suggested that in future studies other regions in Iran perform the same analysis to raise the awareness of this premalignant lesion. Also, the changes in the rate of dentists and health centers and their effect on the rate of referrals can be considered over the years.

## Conclusion

The results of this study showed that in patients referred to Shiraz Dental School, OLP was more frequent in middle-aged women with lower levels of education. Furthermore, there was a relatively good awareness among dentists and caregivers of Fars Province regarding OLP referral for better and more accurate diagnosis and treatment. However, because the disease is classified as a premalignant lesion of the oral cavity, prompt referral, and accurate treatment are necessary. Therefore, both groups (general dentists and health care centers) need to raise awareness about the disease and refer these patients to more specialized centers in Fars, Iran. Moreover, it was seen that referrals from nearby cities to Shiraz are more frequent due to ease of accessibility.

## Acknowledgement

This article is extracted from Dr. Elaheh Zamirian's thesis number 9198327. The authors thank the Vice-Chancellery of Shiraz University of Medical Sciences for supporting this research.

## Conflict of Interests

 The authors declare that they have no conflict of interest.
